# Lactate Profile Assessment—A Good Predictor of Prognosis in Patients with COVID-19 and Septic Shock Requiring Continuous Renal Therapy

**DOI:** 10.3390/clinpract14030078

**Published:** 2024-05-27

**Authors:** Cosmin Iosif Trebuian, Adina Maria Marza, Raul Chioibaş, Dumitru Şutoi, Alina Petrica, Iulia Crintea-Najette, Daian Popa, Florin Borcan, Daniela Flondor, Ovidiu Alexandru Mederle

**Affiliations:** 1 Department of Surgery I, Faculty of Medicine, Victor Babes University of Medicine and Pharmacy, 2nd Eftimie Murgu Square, 300041 Timisoara, Romania; trebuian.cosmin@umft.ro (C.I.T.); marza.adina@umft.ro (A.M.M.); dumitru.sutoi@umft.ro (D.Ş.); alina.petrica@umft.ro (A.P.); iulia.crintea@umft.ro (I.C.-N.); daian-ionel.popa@umft.ro (D.P.); mederle.ovidiu@umft.ro (O.A.M.); 2 Department of Anesthesia and Intensive Care, Emergency County Hospital Resita, 320210 Resita, Romania; 3 Emergency Department, Emergency Clinical Municipal Hospital Timisoara, 300079 Timisoara, Romania; 4 Emergency Department of “Pius Brinzeu”, Emergency Clinical County Hospital Timisoara, 300736 Timisoara, Romania; 5 Faculty of Pharmacy, Victor Babes University of Medicine and Pharmacy, 2nd Eftimie Murgu Square, 300041 Timisoara, Romania; fborcan@umft.ro (F.B.); ionescu.daniela@umft.ro (D.F.); 6 Research Center for Pharmaco-Toxicological Evaluation, Victor Babes University of Medicine and Pharmacy Timisoara, 2nd Eftimie Murgu Square, 300041 Timisoara, Romania

**Keywords:** sepsis, mortality, survival, lactate clearance, acute kidney injury, plasmapheresis, continuous venovenous hemofiltration with dialysis

## Abstract

Introduction: Lactate is a useful prognostic marker, as its level increases in hypoxic tissue and/or during accelerated aerobic glycolysis due to excessive beta-adrenergic stimulation and decreased lactate clearance. The Surviving Sepsis Campaign Bundle 2018 Update suggests premeasurement of lactate within 2–4 h so that physicians perform, assist, administer, and introduce lactate-guided resuscitation to reduce mortality due to sepsis. Methods: A total of 108 patients with septic shock who underwent continuous renal replacement therapy (CRRT) for acute kidney injury were enrolled in this observational study. Demographic, clinical, and laboratory data were collected, and patients were divided into two groups: survivors and non-survivors. Results: Multivariate analysis demonstrated that lactate levels at 24 h after initiation of CRRT treatment, but not lactate levels at intensive care unit (ICU) admission, were associated with mortality. Lactate clearance was associated with lower mortality among the survivors (OR = 0.140) at 6 h after ICU admission and late mortality (OR = 0.260) after 24 h. The area under the ROC curves for mortality was 0.682 for initial lactate; 0.797 for lactate at 24 h; and 0.816 for lactate clearance at 24 h. Conclusions: Our result reinforces that the determination of lactate dynamics represents a good predictor for mortality, and serial lactate measurements may be more useful prognostic markers than initial lactate in patients with septic shock.

## 1. Introduction

Lactate and its clearance are crucial in various medical conditions, including renal support procedures like hemodialysis. Elevated lactate levels are associated with poor outcomes in critically ill patients, such as septic patients with acute kidney injury (AKI), reflecting tissue hypoxia and metabolic stress [1,2]. Continuous renal replacement therapy (CRRT) is used to manage fluid overload, correct electrolyte imbalances, and aid in lactate clearance [3], but, sometimes, it may not always significantly reduce lactate levels, particularly in cases of persistent circulatory ischemia and hypoxia [2]. The relationship between lactate trajectory and mortality in sepsis-associated AKI patients undergoing CRRT has been extensively studied. In critically ill patients, elevated lactate levels have been linked to increased mortality rates, and lactate clearance was used in predicting outcomes. The timing of initiating CRRT is crucial for improving patient outcomes, given that an early initiation of CRRT has been associated with better survival rates and reduced morbidity in this patient population [4]. Additionally, the relationship between the lactate trajectory and 28-day mortality in the ICU demonstrates the potential of CRRT in managing lactate levels and enhancing patient prognosis [2]. Therefore, CRRT is a valuable tool for addressing acid–base and electrolyte imbalances in AKI, further emphasizing its role in optimizing patient care and reducing morbidity [5].

Lactic acidosis, a condition characterized by elevated lactate levels, can result from increased lactate production or impaired lactate clearance [6], being a common complication in sepsis and AKI [3]. Persistent acidosis during CRRT is a strong predictor of poor prognosis, highlighting the importance of lactate normalization in these patients. Although CRRT can correct metabolic acidosis and assist in lactate clearance [3], further investigation is needed to determine the optimal timing and intensity of therapy concerning lactate clearance and morbidity rates to enhance patient management [4]. In refractory cardiogenic shock patients undergoing extracorporeal membrane oxygenation (ECMO), rising lactate levels have been associated with postoperative renal failure and poor outcomes [7]. Lactate clearance during ECMO therapy can affect outcomes, especially in pediatric patients [8]. Furthermore, impaired lactate clearance has been linked to poor prognosis in sepsis and cardiogenic shock [9,10]. In hemodialysis patients, the relationship between lactate/lactic clearance and morbidity rates becomes significant. Reduced lactate clearance may indicate globally impaired renal and hepatic metabolic function, which can contribute to systemic lactate accumulation [11,12]. Studies have suggested that lactate clearance serves as a marker for ongoing tissue hypoxia and can predict mortality in patients with severe sepsis [13,14]. Additionally, improving lactate clearance has been associated with better outcomes in patients with sepsis and AKI [15,16]. In the setting of cardiac surgery and extracorporeal life support (ECLS), elevated lactate levels and impaired lactate clearance have been linked to poor outcomes, emphasizing the importance of monitoring lactate dynamics [17,18]. It was stated that a lactate clearance of less than 10% at 24 h has been correlated with increased mortality in children with shock [19], and perioperative hyperlactatemia and decreased lactate clearance were identified as potential predictors for various infections after cardiac surgery [20].

In conclusion, the prescription of CRRT is a pathway to optimize therapy in critically ill patients, providing a tailored approach to managing conditions like sepsis and AKI [21]. Recent advancements in CRRT techniques, such as the use of membrane adsorbers for endotoxin and cytokines, have shown promising results in improving patient outcomes [21]. Understanding the impact of CRRT on lactate levels and clearance in septic patients is crucial for comprehending the relationship between lactate metabolism and morbidity rates [2]. Additionally, using CRRT in critical care settings significantly contributes to managing renal function and addressing complications associated with AKI [22].

Therefore, the interplay between lactate metabolism, CRRT, and morbidity rates in critically ill patients receiving renal assistance is a complex area that necessitates comprehensive evaluation and management strategies. Monitoring lactate levels, optimizing CRRT protocols, and early initiation of therapy are essential components for reducing morbidity in these vulnerable populations. Further research and clinical studies are needed to elucidate the intricate mechanisms underlying lactate clearance, renal support procedures, and their impact on morbidity rates in critically ill patients.

Thus, the present study aims to establish the impact of lactate and its clearance on morbidity rates in septic shock patients undergoing CRRT. We propose to clarify the precise relationship between lactate levels, renal support interventions, and patient outcomes, to prevent possible unnecessary lactate monitoring after the initiation of CRRT.

## 2. Materials and Methods

### 2.1. Consent and Ethics Approval

The ethics committee of Resita County Emergency Hospital (Caras-Severin, Romania) approved the present study, which was conducted in accordance with the Declaration of Helsinki and approved by the ethics committee of Resita County Emergency Hospital (Caras-Severin, Romania), with the protocol code 1424 on 30 January 2019. The individual data of each patient were entered into an Excel file, being stored as an encrypted file, to protect the confidentiality of the patients. Since the collection of patient data from the hospital database did not change their management in any way, and the statistical analyses were processed anonymously, the informed consent of each patient was no longer necessary for participation in this study with a retrospective design.

### 2.2. Study Design and Patient Population

**Figure 1:**
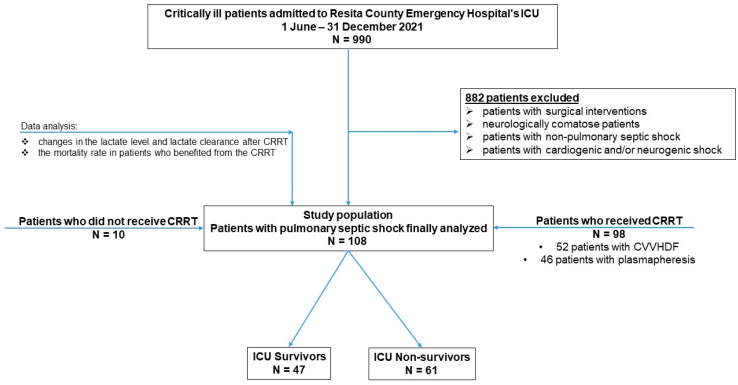
Flow diagram of the analyzed pulmonary septic shock patients.

The present study represents an observational study of a cohort of 990 patients who were admitted to the critical care unit of the Resita County Emergency Hospital (Caras-Severin, Romania, latitude 45°17′09.60″ N, longitude 21°53′30.12″ E), from June 2021 to December 2021. The exclusion criteria referred to patients under the age of 18, those who suffered previous surgical interventions, neurologically comatose patients, patients with non-pulmonary septic shock, or those presenting a state of shock of another etiology (cardiogenic and/or neurogenic) (n = 882). Finally, a total of 108 patients remained as the study population to be analyzed, with age > 18 years and admission to ICU with a diagnosis of pulmonary septic shock—these variables refer to the inclusion criteria. During these seven months, of the 108 patients with septic shock enrolled in this study, 69 of them were diagnosed with COVID-19, and 39 patients did not have COVID-19. In addition, from the total number of patients included in this study, 98 patients needed continuous renal replacement therapy (CRRT) (plasmapheresis and continuous venovenous hemofiltration with dialysis (CVVHDF)). Among them, only 28 patients benefited from both renal support procedures, 52 patients benefited from CVVHDF, and 46 patients benefited only from plasmapheresis (Figure 1).

For the CVVHDF therapy, a Prismaflex System from Baxter International Inc. (Deerfield, IL, USA) was used. This system supports the individual needs of the patients with single or multiple organ failure therapies in one device. An Infomed HF440 CRRT machine from LINC Medical Systems Ltd. (Leicester, UK) was used for plasmapheresis therapy.

### 2.3. Data Collection

Demographic and clinical data including age, gender, virus infection, smoking history, and comorbidities such as chronic heart failure, chronic kidney disease (CKD), chronic obstructive pulmonary disease (COPD), obesity, hypertension (HTN), diabetes, neurologic disease, oncological disease, and hematological disease were collected. One hundred and eight patients with the diagnosis of pulmonary septic shock were enrolled in the present study. Of them, 68 patients were men, divided into two groups: survivors (32 patients (68.1%)) and non-survivors (36 patients (59.0%)). Within the survivor group, 22 patients (46.8%) were infected with COVID-19. We also collected the length of stay (LOS) in the intensive care unit (ICU), one of the most commonly used metrics for quality of care, the oxygen saturation (SpO_2_) at ICU admission, the oxygen pressure in arterial blood (PaO_2_) at 1 h of ICU admission, the fraction of inspired oxygen (FiO_2_), and the partial pressure of carbon dioxide (PaCO_2_), as well as the treatment measures during the ICU stay such as the administration of high-flow nasal cannula (HFNC) therapy, administration of continuous positive airway pressure (CPAP), mechanical ventilation, and continuous renal replacement therapy (CRRT)—plasma exchange and continuous venovenous hemodiafiltration (CVVHDF). At ICU admission, the SOFA score (Sequential Organ Failure Assessment) and APACHE II (acute physiology and chronic health evaluation) were calculated [23,24].

The medical devices used for the HFNC therapy measurement, high-flow O_2_ therapy, invasive and non-invasive mechanical ventilation, and oro-tracheal intubation, as well as monitoring devices, were from Draeger (Lübeck, Germany). For monitoring the patients’ vital functions, the Draeger Infinity C700 was used; for high-flow oxygen therapy and non-invasive mechanical and assisted mechanical ventilation, the Dräger Evita^®^ V800 and Dräger Savina^®^ 300 Select were used. For high-flow nasal cannula (HFNC) therapy, the Airvo™ 2 Nasal High Flow/HFNC System from Fisher & Paykel Healthcare Corporation Limited (Auckland, New Zealand) was used, and the cobas b 123 POC system (from Roche Diagnostics GmbH, Mannheim, Germany) was used for arterial gases and electrolytes.

Clinical laboratory data included routine blood tests, white blood cell (WBC) count, neutrophil ratio, lymphocytes, serum pH, and hemoglobin (Hb); inflammatory indicators, procalcitonin (PCT), fibrinogen, and C-reactive protein (CRP); and all lactates at ICU admission and within 24 h after ICU admission. The complete ABG analysis was performed serially, in the first hour after admission to the ICU, then in the interval 4–6 h after admission, and finally at 24 h; the full blood count, complete coagulogram, and biochemistry analysis were repeated between 12 and 24 h, as well as in the morning of the next day after ICU admission.

Initial serum lactate levels were measured at ICU admission, and we followed the lactate levels at 6 and 24 h from the initial measurement. The lactate clearance rate was defined according to Odom et al. [25], using the following equation:

$$Lactate\;clearance\;rate\;[\%]=\frac{lactate\;initial-lactate\;delayed}{lactate\;initial}\times 100$$

Lactate clearance levels were calculated at 6 and 24 h from ICU admission. An increase in lactate is marked by obtaining a negative value, while obtaining a positive value marks a decrease in lactate. The primary clinical outcome of the present study was the mortality rate of patients who benefited from the CRRT. Secondary outcomes, including changes in lactate levels and lactate clearance, especially in patients who benefited from the CRRT, were registered for all participants.

### 2.4. Treatment Procedure

All patients with pulmonary septic shock were treated according to the standard protocol recommended by the Surviving Sepsis Campaign (SSC) guidelines [26]. Fluid resuscitation was performed in the first hour after ICU admission using 10–20 mL/kgc crystalloid solutions depending on the fluid status of every patient. Arterial blood gases (ABGs) were followed, parameters that correlated with lactate, pH, and excess bases (BEs). Noradrenaline (1 mg/mL, Fresenius Kabi Romania SRL, Brasov, Romania) ± Dobutamine (250 mg from Panpharma SA, Luitré-Dompierre, France) infusions were combined to maintain the mean arterial pressure (MAP) over 65 mmHg. Medium patient ICU admission time from the Emergency Department was 6.5 h (2–12 h).

### 2.5. Statistical Analysis

Data analysis was performed using SPSS Statistics version 27, a software application developed by the IBM Company (Armonk, NY, USA). Descriptive statistical analysis was utilized to summarize and describe the demographic and clinical information. The Kolmogorov–Smirnov test was employed to evaluate the normality of the data, with a null hypothesis that the data originate from a normal distribution. The numerical variables that exhibited normal distributions were characterized by their mean and standard deviation, while the other variables were presented as medians and quartiles. The Mann–Whitney U-test was used to analyze non-parametric variables, while parametric variables were assessed using the parametric *t*-test. The receiver operating characteristic (ROC) curves and optimal threshold values were used to assess the accuracy of the interpretation. Statistical significance was established at a *p*-value threshold of 0.05 and highlighted in bold.

## 3. Results

### 3.1. Baseline Characteristics and Clinical Outcomes

Table 1 depicts the comparison of baseline data and clinical characteristics of the patients according to survivors and non-survivors. The baseline data and the clinical characteristics between the survivor and the non-survivor groups were compared among all patients. The results showed that there was no significant difference between the survivors and non-survivors concerning age, gender, comorbidities (with one exception—the patients who suffer from neurologic disease), smoking, LOS ICU, SpO_2_, PaO_2_, FiO_2_, PaCO_2_, and treatment measures during the ICU stay (HFNC, CPAP, and mechanical ventilation) (*p* > 0.05). There were significantly more patients infected with COVID-19 (47 vs. 22, *p* = 0.009), as well as patients who suffer from neurologic disease (24 vs. 7, *p* = 0.006), in the non-survivor group than in the survivor group. However, there were significantly fewer patients who needed CRRT (plasma exchange (18 vs. 28, *p* = 0.002) and CVVHDF (24 vs. 28, *p* = 0.037)) in the non-survivor than in the survivor group. Regarding the APACHE II score, the interquartile range was the same in both groups of patients, and the SOFA score is under the optimal point for discrimination between mortality and survival in both groups of patients (survivors and non-survivors).

Table 2 depicts a comparison between the clinical outcomes (hematological values) of the survivor patients. The results showed significant differences between the survivor and non-survivor groups concerning the CRP (138 vs. 101, *p* = 0.025), procalcitonin (6.8 vs. 3.2, *p* = 0.006), and lactate levels at ICU admission (4.3 vs. 2.9, *p* = 0.001), after 6 h (3.9 vs. 2.5, *p* < 0.001) and after 24 h of ICU admission (4.8 vs. 2.1, *p* < 0.001), which were significantly higher in the non-survivor group as compared to the survivor group. However, the percentage of lactate clearance calculated at 6 h (0 vs. 0.140, *p* < 0.001) and 24 h (−0.2000 vs. 0.260, *p* < 0.001) of ICU admission was significantly lower in the non-survivor group as compared to the survivor group.

### 3.2. Logistic Regression Analysis

To determine the effect of gender, age, hematological values, and biomarkers related to survival status, univariate and multivariate logistic regression analyses were performed and are presented in Table 3.

Multivariate logistic regression analysis adjusted according to gender, age, and hematological values revealed that patients with increased lactate clearance levels at 24 h after ICU admission had significantly decreased mortality (OR 2.556, 95% CI 1.731–3.775, *p* < 0.001). However, the OR of mortality significantly increased with the CRP value (OR 1.006, 95% CI 1.000–1.012, *p* = 0.044). Moreover, the risk of mortality was almost increased by age (OR 1.039, 95% CI 1.000–1.079, *p* = 0.051).

In addition, a subgroup analysis was conducted on the factors influencing the mortality of patients with pulmonary septic shock (lactate parameter at 6 and 24 h after ICU admission and lactate clearance at 6 and 24 h) who received renal replacement therapy (RRT). The significant univariate indicators presented in Table 1 were included in the multivariate logistic regression analysis (Table 4). A survival analysis (Kaplan–Meier) was used to predict the outcomes strictly related to the evolution of lactate parameters and lactate clearance at 6 and 24 h correlated with mortality in the two groups (plasmapheresis and CVVHDF) of septic shock patients. Of the total number of patients (108), only 46 patients underwent plasmapheresis, and 52 patients needed CVVHDF as an RRT.

The results showed that the mortality of the patients admitted to the ICU with septic shock increases with the increase in the lactate parameters at 6 and 24 h in the group of patients who received plasmapheresis as a renal support therapy. Statistical significance was not observed regarding the lactate parameters at 6 and 24 h in the group of patients who received CVVHDF as a renal support therapy. Therefore, as regards the correlations between the evolution of the lactate parameters at 6 and 24 h with mortality, the null hypothesis is rejected, meaning that there is no difference in the overall survival distributions as regards the two CRRT interventions.

After we performed the statistical analysis to predict the outcomes concerning the correlation of lactate clearance at 6 and 24 h with mortality, the results showed some differences in the overall survival distributions for the patients who benefitted from the two CRRT interventions and the patients who did not but without statistical significance (*p* > 0.05).

### 3.3. Predicting Outcomes with Biomarkers

Table 5 shows the biomarker performance to predict septic shock outcomes based on the cutoff points. The best-performing predictive values for mortality were related to a lactate level at ICU admission of 3.25 mmol/L, with a sensitivity of 67.2% and a specificity of 59.6% (AUC = 0.682, *p* = 0.001, 95% CI: 0.580–0.785) as well as a value of procalcitonin of 4.75 ng/mL, with a sensitivity of 63.9% and a specificity of 63.8% (AUC = 0.655, *p* = 0.006, 95% CI: 0.550–0.760), and a value of CRP of 124.5 mg/dL, with a sensitivity of 62.3% and a specificity of 61.7% (AUC = 0.626, *p* = 0.025, 95% CI: 0.520–0.733).

Slightly lower values, but still high, were also obtained at 6 h and 24 h after ICU admission; at 6 h after ICU admission, the ROC curve showed a value of 2.75 mmol/L for lactate level, with a sensitivity of 68.9% and a specificity of 57.4% (AUC = 0.711, *p* < 0.001, 95% CI: 0.611–0.811), and a value of 2.15 mmol/L at 24 h after ICU admission, with a sensitivity of 77% and a specificity of 53.2% (AUC = 0.797, *p* < 0.001, 95% CI: 0.712–0.883).

The AUC value of the lactate clearance ratio at 6 h was 0.717 (*p* < 0.001, 95% CI: 0.618–0.817), being slightly higher than that of the lactate level at 6 h. The same statistical result was obtained as regards the lactate clearance ratio at 24 h (AUC = 0.816, *p* < 0.001, 95% CI: 0.730–0.902), which was also slightly higher than that of the lactate level at 24 h after ICU admission.

**Figure 2:**
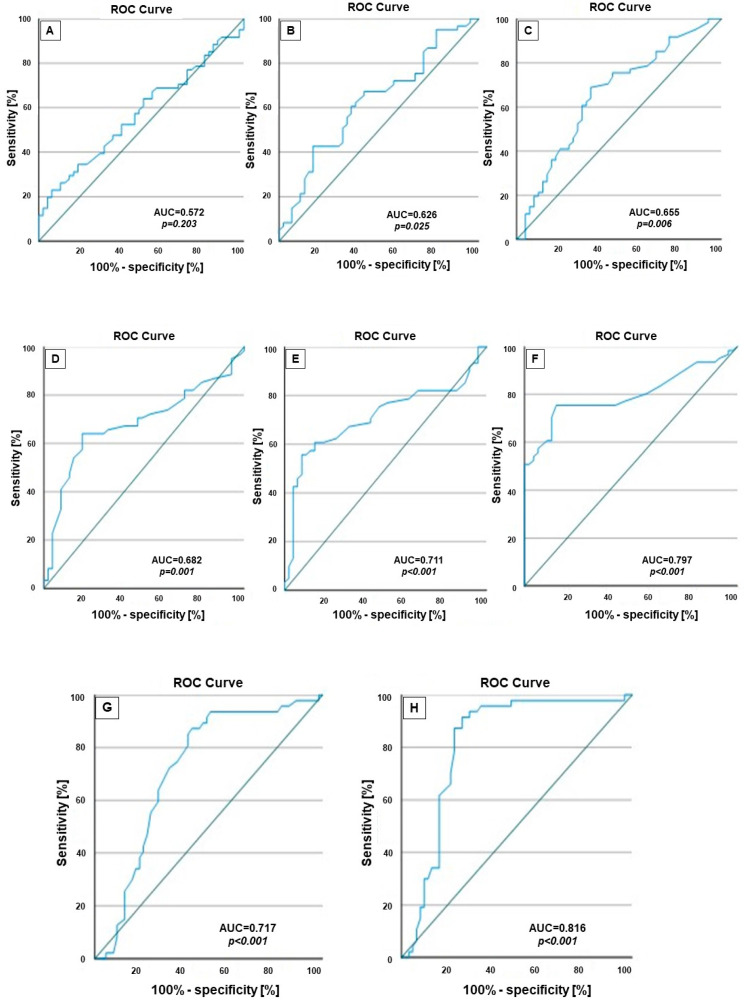
Receiver operating characteristic (ROC) curve analysis for evaluating the accuracy of (**A**) WBCs, (**B**) C-reactive protein, (**C**) procalcitonin, (**D**) lactate level at ICU admission, (**E**) lactate level at 6 h after ICU admission, (**F**) lactate level at 24 h after ICU admission, (**G**) lactate clearance at 6 h, and (**H**) lactate clearance at 24 h in predicting mortality among septic patients.

The ROC curves for the inflammatory biomarkers (WBC, CRP, and procalcitonin), as well as the lactate parameters (lactate level at ICU admission, after 6 h and 24 h, and lactate clearance at 6 h and 24 h) used to predict outcomes (mortality in patients with pulmonary septic shock), are presented in Figure 2.

Table 6 depicts Spearman’s correlations between inflammatory biomarkers, lactate parameters, and survival status. The values presented in Table 6 display only the statistically significant data to provide an effective presentation of the correlations between the parameters studied by our group. This approach prevents an excessively detailed presentation, including parameters such as age, gender, WBCs, CRP, procalcitonin, etc., whose *p* > 0.05 values indicate no significant correlation.

The values highlighted in bold represent the strong correlations between the parameters in the columns and rows, and the rest of the values are moderate correlations. The outcomes showed inverse correlations between the lactate clearance at 6 h and 24 h after ICU admission and mortality (*p* < 0.001), as well as between the lactate clearance at 6 h and 24 h and median lactate at 6 h and 24 h (*p* < 0.001). In addition, the rate of mortality is directly dependent on the lactate parameter at ICU admission (*p* < 0.01) and on the median lactate at 6 h and 24 h (*p* < 0.001). Moreover, fibrinogen is directly dependent on the CRP values (*p* < 0.001).

## 4. Discussion

Septic shock was defined as sepsis-induced hypotension persisting despite adequate fluid resuscitation. Based on Sepsis-3, septic shock was clinically defined as sepsis associated with persisting hypotension, requiring the use of vasopressors to maintain mean arterial pressure (MAP) ≥ 65 mmHg, and serum lactate > 2 mmol/L (18 mg/dL), despite adequate volume resuscitation [27]. Data from recently published trials support this hierarchical stratification, with the mortality from sepsis ranging from 10% to 15%, severe sepsis from 17% to 20%, and septic shock from 43% to 54% [26]. Research studies report that the long-term outcomes of septic shock are poor, e.g., the 6-month mortality of septic shock was 45% [28], and critically ill patients with AKI had a higher 1-year mortality [29].

Understanding the impact of lactate and lactic clearance on morbidity rates in patients undergoing CRRT is essential in critical care management, while CRRT is essential in managing AKI and associated complications. The prognosis of critically ill patients with pulmonary septic shock is very poor, and often these patients are subjected to a rather large economic effort. Therefore, by predicting the outcomes of these patients, clinicians can make the best decisions related to the well-established protocol for personalized treatment and modality.

The present study highlights the clinical utility of lactate levels and lactate clearance as predictive factors of 24 h mortality in critically ill patients with pulmonary septic shock in need of RRT. The statistical analysis concerning the baseline data and clinical parameters between the two groups showed that there were no differences between the survivors and the non-survivors in regard to age, smoking, LOS ICU, SpO_2_, PaO_2_, FiO_2_, PaCO_2_, and treatment measures during the ICU stay (*p* > 0.05). Among the comorbidities recorded in the cohort study, HTN was the most common (69 patients from 108), followed by diabetes, obesity, and neurologic disease. Patients with neurologic disease showed a higher rate of non-survival (*p* = 0.006). Comorbidities like cardiovascular, cerebrovascular, and chronic diseases (pulmonary/kidney) in pulmonary septic shock patients were associated with high long-term mortality in several studies [30,31,32,33]. Regarding pulmonary infections, the patients infected with COVID-19 present an increased rate of mortality (*p* = 0.009). Our findings align with other research studies that discuss the association between pulmonary infection and short-/long-term mortality [34,35].

In addition, the patients who received CRRT (plasmapheresis or CVVHDF) also had a lower survival rate (*p* = 0.002; *p* = 0.037) as compared to the patients who received both CRRTs. In our study, patients with pulmonary septic shock were old (between 62 and 83 years), and their calculated APACHE II scores were quite high; therefore, they were exposed to a higher rate of renal failure. It was stated that older age can be considered a risk factor for mortality among septic shock patients [36]. Another score used for the evaluation and prognosis of sepsis is the SOFA score, which can be determined by assessing the degree of dysfunction of several organs [37]. When it correlates with high mortality rates, its value is high at admission and increased in the first 3 days after ICU admission [38]. In this study, the SOFA scores in both survivor and non-survivor groups were under the optimal point for discrimination between mortality and survival (SOFA = 5). A SOFA score ≥ 3 is correlated with lower survival compared with patients scoring 2 points in our study. More possible prognostic clinical and laboratory factors were analyzed for their influence on mortality (Table 1 and Table 2). Our results suggest that lactate levels at 24 h after CRRT initiation were associated with mortality. Lactate clearance values of over 0.1 (10%) were independently associated with lower mortality among the survivor group (OR (95% CI) = 0.140 (0.09–0.250)) at 6 h after ICU admission and late mortality (OR (95% CI) = 0.260 (0.220–0.360)) after 24 h. Therefore, the lactate clearance, defined by the mean of lactate cleared over 24 h after the initiation of CRRT, was independently associated with lower mortality. In septic shock patients, the lactate clearance over time turned out to be superior to oxygen delivery and consumption [39]. Our results agree with this statement because lactate clearance values over 0.1 were associated with survival. In addition, the results are sustained also by the multivariate logistic regression analysis (Table 3), in which it has been shown that lactate levels at 24 h after the initiation of CRRT treatment, but not lactate levels at ICU admission, were associated with mortality. The subgroup analysis which was performed to predict the outcomes related to the lactate levels and lactate clearance of the patients with septic shock who received CRRT treatment (Table 4) revealed no differences in the overall survival distributions for both CRRT interventions. Analysis of the area under the ROC curve (Table 5 and Figure 2) has shown that the lactate level at 24 h demonstrated better prognostic value than the lactate level at ICU admission. The cutoff value of the lactate level at ICU admission was 3.25 mmol/L, almost identical to the critical lactate value in sepsis (3.0 mmol/L) [40]. Over time, under CRRT treatment, the values of lactate levels decreased. Therefore, our result reinforces the fact that, in sepsis, the determination of lactate dynamics represents a good predictor for mortality. Once again, this study confirms that lactate level and lactate clearance could predict the survival outcome in the case of patients with pulmonary septic shock, in agreement with previous studies [41,42,43].

Although the APACHE II and SOFA scores were the same in both groups (meaning that they did not discriminate the prognosis), the evolution of lactate levels and lactate clearance during CRRT treatment was significantly different between both survivor and non-survivor patients. This finding is correlated with some studies that have shown that the failure of metabolic acidosis correction during the CRRT treatment is also a strong predictor of mortality [44,45]. Future studies should focus on determining the causality of lactate levels and mortality. Our study is a clinical observational study, so therefore causality is less convincing than CRRT treatment. Doctors make decisions based on changes in the patient’s condition; therefore, the relationship between the lactate level and mortality is not a simple causal relationship because, after treatment, high lactate levels may decrease, and in patients with normal lactate levels, doctors may neglect to apply a treatment.

In summary, the predictive ability of lactate levels increased from ICU admission until 24 h; we consider that this was the optimal timing for predicting outcomes. Therefore, patients with pulmonary septic shock in need of CRRT treatment are independently associated with higher odds of death and longer duration of hospitalization. Until now, little was known about risk-stratification biomarkers in the case of septic shock patients who need CRRT, but our data suggest that lactate could be a plausible predictive factor.

Our study has several limitations. The first limitation is related to the nature of the study. This study was a single-center observational one; thus, its design will contain a lack of representativeness. Because of the observational nature, the obtained outcomes are of association, and causation needs further confirmation. Nonetheless, our study was based on a significant correlation between lactate clearance and decreased mortality, agreeing with previous studies on different populations of patients. The second limitation is related to the diagnosis of septic shock that was established during the first 24 h after ICU admission; therefore, the patients who developed septic shock after this were not analyzed. The patients included in our study were older with complications that turned out to be more serious than at ICU admission; consequently, the obtained outcomes related to mortality could be overestimated.

## 5. Conclusions

By elucidating the relationship between lactate dynamics, renal function, and morbidity rates, healthcare providers can better tailor interventions to enhance lactate clearance and mitigate associated risks. The use of lactate and its clearance as predictors of outcome, as markers to initiate therapy, and to monitor the adequacy of initiated treatments, using measurements of lactate parameters at 1–6–24 h and lactate clearance (between 1 and 6 h and 6 and 24 h) in pulmonary septic shock patients, was described. Lactate levels at 24 h after initiation of CRRT treatment and lactate clearance were associated with 24 h mortality in pulmonary septic shock patients who underwent CRRT. Our study confirms once again the association between the lactate level and mortality rate in patients with pulmonary septic shock, but further studies are needed to determine the causality between lactate levels and mortality.

## Figures and Tables

**Table 1:** Demographic and clinical characteristics of participants according to survivor and non-survivor groups.

Variables	Survivors (n = 47)	Non-Survivors (n = 61)	*p*-Value
Age (range, years)	71 (62–81)	75 (63–82.5)	0.357
Male % (n)	68.1 (32)	59.0 (36)	0.333
COVID-19 infection % (n)	46.8 (22)	77 (47)	0.009
Comorbidities % (n)			
Chronic heart failure	21.3 (10)	36.1 (22)	0.095
CKD	10.6 (5)	14.8 (9)	0.577
COPD	23.4 (11)	16.4 (10)	0.361
Obesity	40.4 (19)	32.8 (20)	0.413
HTN	59.6 (28)	67.2 (41)	0.413
Diabetes	51.1 (24)	42.6 (26)	0.383
Neurologic disease	14.9 (7)	39.3 (24)	0.006
Oncological disease	10.6 (5)	6.6 (4)	0.499
Hematological disease	36.2 (17)	29.5 (18)	0.500
Smoking % (n)	17 (8)	13.3 (8)	0.599
LOS ICU (range, days)	7 (6–10)	6 (4–10)	0.083
Plasma exchange % (n)	59.6 (28)	30 (18)	0.002
CVVHDF % (n)	59.6 (28)	39.3 (24)	0.037
HFNC % (n)	68.1 (32)	65.6 (40)	0.784
CPAP % (n)	55.3 (26)	58.3 (35)	0.755
Mechanical ventilation % (n)	55.3 (26)	63.3 (38)	0.401
SpO_2_ at ICU admission (%)	81 (74–87)	78 (74–88)	0.377
PaO_2_ at 1 h of ICU admission (mmHg) (range, value)	56 (49–62)	54 (47–61.5)	0.511
FiO_2_ (%)	1.0 (0.9–1.0)	1.0 (0.9–1.0)	0.997
PaCO_2_ (mmHg) (range, value)	35.4 (29.0–39.0)	36 (29–42.9)	0.671
APACHE II score	27 (23–35)	27 (23–35)	<0.001
SOFA score	2 (1–3)	2 (1–3)	<0.001

CKD—chronic kidney disease; COPD—chronic obstructive pulmonary disease; HTN—hypertension; LOS ICU—length of stay in the intensive care unit; CVVHDF—continuous venovenous hemodiafiltration; HFNC—high-flow nasal cannula; CPAP—continuous positive airway pressure; SpO_2_—peripheral capillary oxygen saturation; PaO_2_—partial pressure of oxygen in arterial blood; FiO_2_—fraction of inspired oxygen; PaCO_2_—partial pressure of carbon dioxide in arterial blood.

**Table 2:** Hematological comparison by survival status.

Variables	Survivors	Non-Survivors	*p*-Value
WBC × 10^3^/mm^3^	16.03 (11.36, 21.58)	18.36 (11.88, 26.28)	0.203
Neutrophils × 10^3^/mm^3^	11.77 (1.87, 17.48)	10.25 (3.80, 17.56)	0.463
Lymphocytes × 10^3^/mm^3^	0.500 (0.106, 1.198)	0.533 (0.123, 1.871)	0.319
C-reactive protein mg/dL	101 (59, 154)	138 (69.5, 207.5)	0.025
Procalcitonin ng/mL	3.2 (0.9, 8.6)	6.8 (3.3, 10.8)	0.006
Fibrinogen g/L	419 (275, 559)	460 (287, 621)	0.381
Serum pH	7.43 (7.35, 7.48)	7.39 (7.29, 7.47)	0.270
Hb g/L	13.5 (11.1, 14.7)	13.0 (11.2, 14.7)	0.901
Lactic acid at ICU admission mmol/L	2.9 (2.4, 3.6)	4.3 (2.7, 4.8)	0.001
Mean lactate at 6 h mmol/L	2.5 (2.1, 3.1)	3.9 (2.55, 5.0)	<0.001
Mean lactate at 24 h mmol/L	2.1 (1.8, 2.5)	4.8 (2.5, 5.85)	<0.001
Lactate clearance at 6 h of ICU admission	0.140 (0.09, 0.250)	0 (−0.090, 0.1350)	<0.001
Lactate clearance at 24 h of ICU admission	0.260 (0.220, 0.360)	−0.2000 (−0.2750, 0.1500)	<0.001

WBC, white blood cell; Hb, hemoglobin.

**Table 3:** Regression analysis of factors related to survival status.

Variables	Univariate Analysis		Multivariate Analysis	
	OR (95% CI)	*p*-Value	OR (95% CI)	*p*-Value
Mortality (yes vs. no)				
Gender	0.675 (0.304–1.499)	0.334	0.753 (0.270–2.102)	0.588
Age	1.017 (0.990–1.045)	0.229	1.039 (1.000–1.079)	0.051
WBC	1.036 (0.988–1.086)	0.144	1.003 (0.933–1.078)	0.940
CRP	1.005 (1.001–1.010)	0.029	1.006 (1.000–1.012)	0.044
Procalcitonin	1.036 (0.986–1.088)	0.157	0.996 (0.938–1.059)	0.905
Fibrinogen	1.001 (0.999–1.003)	0.346	1.000 (0.996–1.003)	0.790
Lactic acid at ICU admission	1.587 (1.145–2.201)	0.006		
Lactic acid at 6 h after ICU admission	1.954 (1.376–2.776)	<0.001		
Lactic acid at 24 h after ICU admission	2.440 (1.687–3.528)	<0.001		
Lactate clearance at 6 h	0.058 (0.007–0.476)	0.008		
Lactate clearance at 24 h	0.011 (0.002–0.065)	<0.001	2.556 (1.731–3.775)	<0.001

OR—odds ratio; CI—confidence interval.

**Table 4:** Multivariate analysis of lactate parameters and lactate clearance at 6 and 24 h for mortality of patients with pulmonary septic shock who benefited from plasmapheresis and CVVHDF therapy support.

Variables	RRT Instituted	No. of Events	No. of Patients	Censored		Mortality [%] ^a^	Chi-Square	*p*-Value
				N	Percent	(95% CI)		
Lactate parameter at 6 and 24 h after ICU admission	Plasmapheresis						11.16	0.001
	With	18	46	28	60.9%	15.65 (12.25–19.05)		
	Without	43	62	19	30.6%	4.07 (0.52–7.61)		
	Overall	61	108	47	43.5%	8.91 (5.52–12.29)		
	CVVHDF						3.45	0.063
	With	24	52	28	53.8%	10.04 (4.16–15.92)		
	Without	37	56	19	33.9%	7.66 (3.71–11.62)		
	Overall	61	108	47	43.5%	8.91 (5.52–12.29)		
Lactate clearance at 6 and 24 h	Plasmapheresis						2.14	0.143
	With	18	46	28	60.9%	19.70 (17.41–21.98)		
	Without	43	62	19	30.6%	17.61 (15.44–19.78)		
	Overall	61	108	47	43.5%	18.50 (16.92–20.08)		
	CVVHDF						2.97	0.085
	With	24	52	28	53.8%	19.5 (17.34–21.66)		
	Without	37	56	19	33.9%	17.57 (15.28–19.86)		
	Overall	61	108	47	43.5%	18.50 (16.92–20.08)		

^a^—the mortality is expressed as a mean estimation which is limited to the largest survival time if it is censored.

**Table 5:** Comparison of inflammatory biomarkers and lactate parameters in discriminating survival among septic patients.

	AUC	SE	95% CI	Sensitivity	Specificity	Cutoff Points	*p*-Value
WBC	0.572	0.055	0.464–0.679	0.541	0.532	16.705	0.203
CRP	0.626	0.054	0.520–0.733	0.623	0.617	124.5	0.025
Procalcitonin	0.655	0.054	0.550–0.760	0.639	0.638	4.75	0.006
Lactate level at ICU admission	0.682	0.052	0.580–0.785	0.672	0.596	3.25	0.001
Lactate level at 6 h after ICU admission	0.711	0.051	0.611–0.811	0.689	0.574	2.75	<0.001
Lactate level at 24 h after ICU admission	0.797	0.044	0.712–0.883	0.770	0.532	2.15	<0.001
Lactate clearance at 6 h	0.717	0.051	0.618–0.817	0.638	0.721	0.1150	<0.001
Lactate clearance at 24 h	0.816	0.044	0.730–0.902	0.783	0.770	0.2150	<0.001

AUC—area under the curve; SE—Standard Error; 95% CI—95% confidence interval.

**Table 6:** Spearman’s correlations between inflammatory biomarkers, lactate parameters, and survival status.

Variable		Death	Lactate at ICU Admission	Median Lactate at 6 h	Median Lactate at 24 h	Lactate Clearance at 6 h	Lactate Clearance at 24 h	CRP
Death	Spearman’s rho	-						
	*p*-value	-						
Lactate at ICU admission	Spearman’s rho	0.314 ***	-					
	*p*-value	<0.001	-					
Median lactate at 6 h	Spearman’s rho	0.363 ***	0.818 ***	-				
	*p*-value	<0.001	<0.001	-				
Median lactate at 24 h	Spearman’s rho	0.511 ***	0.756 ***	0.896 ***	-			
	*p*-value	<0.001	<0.001	<0.001	-			
Lactate clearance at 6 h	Spearman’s rho	−0.373 ***	-	−0.512 ***	−0.497 ***	-		
	*p*-value	<0.001	-	<0.001	<0.001	-		
Lactate clearance at 24 h	Spearman’s rho	−0.543 ***	-	−0.462 ***	−0.668 ***	0.772 ***	-	
	*p*-value	<0.001	-	<0.001	<0.001	<0.001	-	
FBG	Spearman’s rho	-	-	-	-	-	-	0.393 ***
	*p*-value	-	-	-	-	-	-	˂0.001

***—*p* < 0.001; FBG—fibrinogen.

## Data Availability

The corresponding author can provide all data collected and analyzed in the present study, upon reasonable request.

## References

[1] Bellomo R. (2002). Bench-to-bedside review: Lactate and the kidney. Crit. Care.

[2] Wang Z., Zhang L., Xu F., Han D., Lyu J. (2022). The association between continuous renal replacement therapy as treatment for sepsis-associated acute kidney injury and trend of lactate trajectory as risk factor of 28-day mortality in intensive care units. BMC Emerg. Med..

[3] Passos R.D.H., Ramos J.G.R., Gobatto A., Mendonça E.J.B., Miranda E.A., Dutra F.R.D., Coelho M.F.R., Pedroza A.C., Batista P.B.P., Dutra M.M.D. (2016). Lactate clearance is associated with mortality in septic patients with acute kidney injury requiring continuous renal replacement therapy: A cohort study. Medicine.

[4] Yoon B.R., Leem A.Y., Park M.S., Kim Y.S., Chung K.S. (2019). Optimal timing of initiating continuous renal replacement therapy in septic shock patients with acute kidney injury. Sci. Rep..

[5] Yessayan L., Yee J., Frinak S., Szamosfalvi B. (2016). Continuous renal replacement therapy for the management of acid-base and electrolyte imbalances in acute kidney injury. Adv. Chronic Kidney Dis..

[6] Possemiers H., Vandermosten L., Van den Steen P. (2021). Etiology of lactic acidosis in malaria. PLoS Pathog..

[7] Khorsandi M., Dougherty S., Bouamra O., Pai V., Curry P., Tsui S., Clark S., Westaby S., Al-Attar N., Zamvar V. (2017). Extra-corporeal membrane oxygenation for refractory cardiogenic shock after adult cardiac surgery: A systematic review and meta-analysis. J. Cardiothorac. Surg..

[8] Merkle-Storms J., Djordjevic I., Weber C., Avgeridou S., Krasivskyi I., Gaisendrees C., Mader N., Kuhn-Régnier F., Kröner A., Bennink G. (2021). Impact of Lactate Clearance on Early Outcomes in Pediatric ECMO Patients. Medicina.

[9] Preiser J.C., Provenzano B., Mongkolpun W., Halenarova K., Cnop M. (2020). Perioperative Management of Oral Glucose-lowering Drugs in the Patient with Type 2 Diabetes. Anesthesiology.

[10] Mathew S., Whitman L. (2020). 206. The utility of lactate as a biomarker for sepsis in cancer patients. Open Forum Infectious Diseases.

[11] Mungan İ., Kazancı D., Bektaş Ş., Ademoğlu D., Turan S. (2018). Does lactate clearance prognosticates outcomes in ECMO therapy: A retrospective observational study. BMC Anesthesiol..

[12] Verissimo T., Faivre A., Rinaldi A., Lindenmeyer M., Delitsikou V., Veyrat-Durebex C., Heckenmeyer C., Fernandez M., Berchtold L., Dalga D. (2022). Decreased renal gluconeogenesis is a hallmark of chronic kidney disease. J. Am. Soc. Nephrol..

[13] Shadvar K., Nader N., Vahed N., Sanaie S., Iranpour A., Mahmoodpoor A., Vahedian-Azimi A., Samim A., Rahimi-Bashar F. (2022). Comparison of lactate/albumin ratio to lactate and lactate clearance for predicting outcomes in patients with septic shock admitted to intensive care unit: An observational study. Sci. Rep..

[14] Bhat S., Swenson K., Francis M., Wira C. (2015). Lactate clearance predicts survival among patients in the emergency department with severe sepsis. West. J. Emerg. Med..

[15] Chertoff J., Chisum M., Simmons L., King B., Walker M., Lascano J. (2016). Prognostic utility of plasma lactate measured between 24 and 48 h after initiation of early goal-directed therapy in the management of sepsis, severe sepsis, and septic shock. J. Intensive Care.

[16] He M., Huang J., Li X., Liang S., Wang Q., Zhang H. (2023). Risk factors for mortality in sepsis patients without lactate levels increasing early. Emerg. Med. Int..

[17] Nicola R., Shaqdan K., Aran K., Mansouri M., Singh A., Abujudeh H. (2015). Contrast-induced nephropathy: Identifying the risks, choosing the right agent, and reviewing effective prevention and management methods. Curr. Probl. Diagn. Radiol..

[18] Scolari F., Schneider D., Fogazzi D., Gus M., Rover M., Bonatto M., Neves de Araújo G., Zimerman A., Sganzerla D., Adams Goldraichet L. (2020). Association between serum lactate levels and mortality in patients with cardiogenic shock receiving mechanical circulatory support: A multicenter retrospective cohort study. BMC Cardiovasc. Disord..

[19] Rissel R., Koelm S., Schepers M., Dohle D., Albers J., Oezkur M., Kriege M., Bodenstein M. (2022). Elevated lactate levels and impaired lactate clearance during extracorporeal life support (ecls) are associated with poor outcome in cardiac surgery patients. PLoS ONE.

[20] Morse B., Vijay N., Morris M. (2014). Mechanistic modeling of monocarboxylate transporter-mediated toxicokinetic/toxicodynamic interactions between γ-hydroxybutyrate and l-lactate. AAPS J..

[21] Karkar A., Ronco C. (2020). Prescription of crrt: A pathway to optimize therapy. Ann. Intensive Care.

[22] Gemmell L., Docking R., Black E. (2017). Renal replacement therapy in critical care. BJA Educ..

[23] Knaus W.A., Draper E.A., Wagner D.P., Zimmerman J.E. (1985). APACHE II: A severity of disease classification system. Crit. Care Med..

[24] Moreno R., Vincent J.L., Matos R., Mendonça A., Cantraine F., Thijs L., Takala J., Sprung C., Antonelli M., Bruining H. (1999). The use of maximum SOFA score to quantify organ dysfunction/failure in intensive care. Results of a prospective, multicentre study. Working group on sepsis related problems of the ESICM. Intensive Care Med..

[25] Odom S.R., Howell M., Silva G., Nielsen V., Gupta A., Shapiro N., Talmor D. (2013). Lactate clearance as a predictor of mortality in trauma patients. J. Trauma. Acute Care Surg..

[26] Rhodes A., Evans L., Alhazzani W., Levy M., Antonelli M., Ferrer R., Kumar A., Sevransky J., Sprung C., Nunnally M. (2017). Surviving sepsis campaign: International guidelines for management of sepsis and septic shock: 2016. Intensive Care Med..

[27] Bruno R.R., Wernly B., Binneboessel S., Baldia P., Duse D.A., Erkens R., Kelm M., Mamandipoor B., Osmani V., Jung C. (2020). Failure of Lactate Clearance Predicts the Outcome of Critically Ill Septic Patients. Diagnostics.

[28] Nesseler N., Defontaine A., Launey Y., Morcet J., Mall’edant Y., Seguin P. (2013). Long-term mortality and quality of life after septic shock: A Follow-Up Observational Study. Intensive Care Med..

[29] Harris D.G., McCrone M.P., Koo G., Weltz A.S., Chiu W.C., Scalea T.M., Diaz J.J., Lissauer M.E. (2015). Epidemiology and outcomes of acute kidney injury in critically ill surgical patients. J. Crit. Care.

[30] Chin Y.R., Lee I.S., Lee H.Y. (2014). Effects of hypertension, diabetes, and/or cardiovascular disease on health-related quality of life in elderly Korean individuals: A population-based cross-sectional survey. Asian Nurs. Res..

[31] Persson J., Holmegaard L., Karlberg I., Redfors P., Jood K., Jern C., Blomstrand C., Forsberg-Wärleby G. (2015). Spouses of stroke survivors report reduced health-related quality of life even in long-term follow-up: Results from Sahlgrenska Academy study on ischemic stroke. Stroke.

[32] Sundh J., Johansson G., Larsson K., Linden A., Löfdahl C.G., Janson C., Sandström T. (2015). Comorbidity and health-related quality of life in patients with severe chronic obstructive pulmonary disease attending Swedish secondary care units. Int. J. Chronic Obstr. Pulm. Dis..

[33] Lee S.J., Son H., Shin S.K. (2015). Influence of frailty on health-related quality of life in pre-dialysis patients with chronic kidney disease in Korea: A cross-sectional study. Health Qual. Life Outcomes.

[34] Kim W.Y., Lee Y.J., Lim S.Y., Koh S.O., Choi W.I., Kim S.C., Chon G.R., Kim J.H., Kim J.Y., Lim J. (2013). Clinical characteristics and prognosis of pneumonia and sepsis: Multicenter Study. Minerva Anestesiol..

[35] Mansur A., Klee Y., Popov A.F., Erlenwein J., Ghadimi M., Beissbarth T., Bauer M., Hinz J. (2015). Primary bacteremia is associated with a higher mortality risk compared with pulmonary and intra-abdominal infections in patients with sepsis: A prospective observational cohort study. BMJ Open.

[36] Drumheller B.C., Agarwal A., Mikkelsen M.E., Sante S.C., Weber A.L., Goyal M., Gaieski D.F. (2016). Risk factors for mortality despite early protocolized resuscitation for severe sepsis and septic shock in the emergency department. J. Crit. Care.

[37] Sales Júnior J.A.L., David C.M., Hatum R., Souza P.C.S., Japiassú A., Pinheiro C.T., Friedman G., Silva O.B.D., Dias M.D.A., Koterba E. (2006). Sepse Brasil: Estudo epidemiológico da sepse em Unidades de Terapia Intensiva brasileiras. Rev. Bras. Ter. Intensiv..

[38] Garnacho-Montero J., Garcia-Garmendia J.L., Barrero-Almodovar A., Jimenez-Jimenez F.J., Perez-Paredes C., Ortiz-Leyba C. (2003). Impact of adequate empirical antibiotic therapy on the outcome of patients admitted to the intensive care unit with sepsis. Crit. Care Med..

[39] Bakker J., Coffernils M., Leon M., Gris P., Vincent J.-L. (1991). Blood lactate levels are superior to oxygen-derived variables in predicting outcome in human septic shock. Chest.

[40] Singer M., Deutschman C.S., Seymour C.W., Shankar-Hari M., Annane D., Bauer M., Bellomo R., Bernard G.R., Chiche J.-D., Coopersmith C.M. (2016). The third international consensus definitions for sepsis and septic shock (Sepsis-3). JAMA.

[41] Slottosch I., Liakopoulos O., Kuhn E., Scherner M., Deppe A.-C., Sabashnikov A., Mader N., Choi Y.-H., Wippermann J., Wahlers T. (2017). Lactate and lactate clearance as valuable tool to evaluate ECMO therapy in cardiogenic shock. J. Crit. Care.

[42] Ryoo S.M., Lee J., Lee Y.-S., Lee J.H., Lim K.S., Huh J.W., Hong S.-B., Lim C.-M., Koh Y., Kim W.Y. (2018). Lactate level versus lactate clearance for predicting mortality in patients with septic shock defined by sepsis-3. Crit. Care Med..

[43] Takahashi N., Nakada T.A., Walley K.R., Russell J.A. (2021). Significance of lactate clearance in septic shock patients with high bilirubin levels. Sci. Rep..

[44] Page B., Vieillard-Baron A., Chergui K., Peyrouset O., Rabiller A., Beauchet A., Aegerter P., Jardin F. (2005). Early veno-venous haemodiafiltration for sepsis-related multiple organ failure. Crit. Care.

[45] Kawarazaki H., Uchino S., Tokuhira N., Ohnuma T., Namba Y., Katayama S., Toki N., Takeda K., Yasuda H., Izawa J. (2013). Who may not benefit from continuous renal replacement therapy in acute kidney injury?. Hemodial. Int..

